# Changes in Plasma and Ovarian Steroid Hormone Level in Wild Female Blue Tang Fish *Paracanthurus hepatus* during a Reproductive Cycle

**DOI:** 10.3390/ani9110889

**Published:** 2019-11-01

**Authors:** Huynh Minh Sang, Ho Son Lam, Le Ho Khanh Hy, Pham Xuan Ky, Phan Minh-Thu

**Affiliations:** 1 Institute of Oceanography, Vietnam Academy of Science and Technology, 01- Cau Da, Nha Trang 650000, Vietnam; hslamqt@gmail.com (H.S.L.); lehokhanhhy@gmail.com (L.H.K.H.); phamkx@vnio.org.vn (P.X.K.); 2 Graduate University of Science and Technology, Vietnam Academy of Science and Technology, 18 Hoang Quoc Viet, Cau Giay, Ha Noi 100000, Vietnam

**Keywords:** steroid hormones, ELISA, wild blue tang, ovarian development, GSI

## Abstract

**Simple Summary:**

Live blue tang fish from Khanh Hoa to Binh Thuan seawaters (a central marine region of Vietnam) were collected monthly for the duration of 12 months and the levels of testosterone (T), estradiol-17β (E_2_) and 17α, 20β-dihydroxy-4-pregnen-3-one (DHP) in the plasma and the gonad; gonadosomatic index were determined. The gonadosomatic index (GSI%) maintained high values from April to July and increased from the II to IV ovarian stages and dropped in the V stage. Levels of plasma and ovary T and E_2_ and DHP were high from March to July. Plasma T and E_2_ levels were low in the II stage when most oocytes were previtellogenic, reaching a peak during spawning and decreased in the V stage when fish were going to late spawning and termination.

**Abstract:**

This study aimed to document the seasonal cycle of steroid levels in the plasma and ovary, including testosterone (T), estradiol-17β (E_2_) and 17α, 20β-dihydroxy-4-pregnen-3-one (DHP) in relation to ovarian development in wild populations of female blue tang fish. The gonadosomatic index (GSI%) maintained high values from April to July and increased from the II to IV ovarian stages and dropped in the V stage. Levels of plasma, ovary T and E_2_ and DHP were high from March to July. Plasma T and E_2_ levels were low in the II stage when most oocytes were previtellogenic, reaching a peak during spawning, and decreased in the V stages when fish were going to late spawning and termination. DHP was detected in all stages of the ovary with a higher level in spawning fish but decreased after spawning. These results revealed that T, E_2_ and DHP could be involved in ovarian development and DHP may play a significant role as a maturational inducing hormone in blue tang fish.

## 1. Introduction

Steroid hormones (such as 17β-estradiol (E_2_), testosterone (T), 11-ketotestosterone (11-KT) and 17α20β- dihydroxy-4-pregnen-3-one (DHP)) play a vital role in the normal gonadal functions in teleosts [1]. Levels of several steroid hormones in gonad and plasma are useful indicators of steroidogenic secretion in association with control of the reproduction in teleosts [2,3,4]. Changes in plasma steroid hormone levels and their possible role in the regulation of gonadal development in some wild and reared teleosts have been discussed. Recent studies have proved that some marine fish are multiple spawning fish. Their ovaries display asynchronous development, in which a final oocyte maturation (FOM) and ovulation can complete within 24 h [5,6,7]. The processes of oocyte growth and FOM and thus distinct steroidal requirements simultaneously take place in the ovary. Therefore, steroid hormones in a multiple spawning fish may have a seasonal and diurnal rhythm, in distinction to fish showing synchronous ovarian development. However, such information is limited in a few daily spawning fish [2,3].

Blue tang fish *Paracanthurus hepatus*, known as a species of Indo-Pacific surgeonfish, is the only member of the genus Paracanthurus. This species is naturally distributed in the waters from East Africa to Micronesia, Line Islands and Samoa Islands, northwards to Kochi Prefecture and southwards to New South Wales, Australia [8]. Despite its broad range, it is common nowhere and can be seen throughout the Indo-Pacific. Blue tang is reported to be an inhabitant in the reef waters in subtropical and tropical marine regions [8,9]. The blue tang is considered as one of the most normal and most popular marine ornamental fish worldwide [10]. They have behavior as living in pairs, or in small groups of 8 to 14 individuals. The fish are generally matured at 9–12 months of age [11]. Recently elevated demand of the blue tang as an aquarium fish has been negatively affecting their natural population in the important coral reefs of central marine regions of Vietnam [9]. Thus, it is urgent to seek the appropriate management policy for natural resource conservation as well as the techniques for captive breeding of this fish as supply for the aquarium industry, alleviating the pressure on natural resources. One of the effective solutions to diminish the natural exploitation is breeding and rearing the fish in captured conditions to provide for the market. Some studies had been carried out to evaluate the wild population of blue tang in Khanh Hoa seawater and to provide the scientific base for artificial breeding of the fish [9,12]. The studies recommended that no more than 1000 individuals of blue tang should be collected annually from Khanh Hoa waters [12]. In nature, gonadal maturation of the fish is categorized into five stages. Research on gonad histology, maturation ratios and gonadal somatic index value of this fish revealed that blue tang is an all-year-round spawning species with the female’s peak spawning occuring from April to September. Blue tang reaches its first sexual maturity when its total length is approximated to be 15 cm. The absolute fecundity had a relatively high correlation to their weight and length of fish [9]. Broodstock of blue tang can be successfully conditioned in capture conditions but breeding stimulation has not been successful yet [12]. Broodstock conditioning is one of the most important parts in the artificial breeding of fish. For the success of broodstock conditioning, knowledge on reproductive endocrinology is needed to provide the scientific foundation for interfering in the maturation and breeding process. However, evidence of reproductive endocrinology in blue tang fish is still lacking. Therefore, the present study aims to investigate the variations in steroid hormones of the levels of plasma and ovaries in relation with ovarian oocyte maturation during one year of the reproductive cycle of blue tang fish.

## 2. Materials and Methods

The care and use of experimental animals complied with Vietnamese animal welfare laws, guidelines and policies as approved by national permitting authority number 26/2019/NĐ-CP issued on 08/03/2019.

### 2.1. Sample Collection

**Figure 1:**
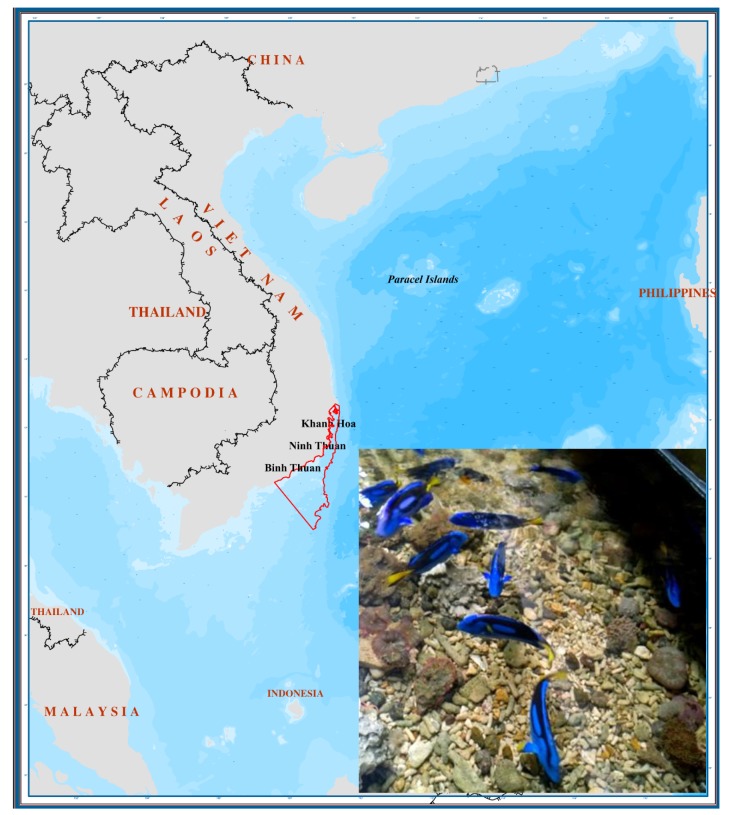
Studied map. Red boundary indicates the reefs from Khanh Hoa to Binh Thuan seawaters where live blue tang fish were collected. The small picture shows fish kept in the aquarium station at the Institute of Oceanography, Vietnam.

Animal: Live blue tang fish (average length: 188.23 ± 33.34 mm) were collected in the coral reefs located in Khanh Hoa–Binh Thuan seawaters, spanning from 12°48.22′ N to 9°33.88′ N and from 107°35.10′ E to 109°27.12′ E (a central marine regions of Vietnam—Figure 1) for the duration of 12 months. About 15 female fish were collected every month by diving fishermen, kept in seawater aeration with oxygen and transported to the aquarium station at the Institute of Oceanography, Vietnam within 12 h. The fish were kept for three days at the aquarium station (Figure 1, small picture).

Sampling: The fish was anesthetized by immersion in ice-cold water and total length and body weight were measured. Its blood and gonads were collected.

Blood sampling: A sample of 0.5–1 mL of the blood of each female was collected by heparinized syringes via the caudal arteries and plasma was separated by centrifugation at 4 °C for 15 min at 1000× *g* and then stored at −35 °C until extraction of steroid hormones.

Gonad sampling: Gonads of the fish were removed to measure the weight. A piece of the gonad was used for histological analysis and another part was stored at −35 °C until the extraction of steroid hormones.

### 2.2. Sample Analysis and Data Collection

Gonadal development stages: Gonadal development stages of the fish were determined by the method described by [13] and Table 1. Almost all female fish collected from January to December were in the II–IV stages. Most fish in the ovarian stages III and IV were observed from March to August.

Extraction of steroid hormone from plasma: The steroid hormone was extracted using diethyl ether. After being completely dried, the extracts were reconstituted in ELISA buffer (MyBioSource, Inc., San Diego, CA, USA) and then stored at −35 °C until assay.

Extraction of steroid hormones from the gonad: The gonad was homogenized and steroid hormones were extracted with 20 mM Tris-buffered NaCl (0.9%) (Sigma-Aldrich, Inc., Saint Louis, MO, USA), pH 7.4 at 5 °C, then centrifuged at 25,000× *g* for 15 min. The solid was discarded and the supernatant fluid was collected for steroid extraction.

Determination of steroid reproductive hormone levels: The commercial ELISA kits to determine T (MBS165765), E2 (MBS70019) in fish were purchased from MyBioSource, Inc (San Diego, CA, USA). and commercial ELISA for 17,20-DHP in fish was from Cayman Chemical (Ann Arbor, MI, USA). ELISA assays for measuring steroid reproductive hormone levels were performed according to the protocol described by the manufacturer.

Evaluating the variation of steroid reproductive hormones: Hormone steroids of the natural caught blue tang fish were evaluated base on the variation in the levels by month and by gonadal development stages and the gonadosomatic index (GSI).

### 2.3. Data Analysis

Hormone steroids of the natural caught blue tang fish was evaluated base on the variation in the levels by gonadal development stages. Before applying the one way analysis of variance (ANOVA) test, all data were checked for normality and homogeneity of variances by the box-whisker diagram and Levene’s test, respectively. The normality data were subjected to the least significant difference (LSD) post hoc test to determine the significant difference between the level of steroid hormones at different ovarian development stages. The level of statistically significant difference was *p* ≤ 0.05. SPSS version 10 (SPSS Inc., Chicago, IL, USA) was used to analyze the data.

## 3. Results

### 3.1. Monthly Change in GSI of Wild-Caught Blue Tang Fish

**Figure 2:**
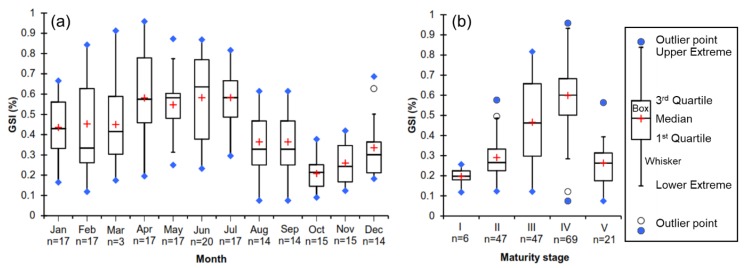
Gonadosomatic index (GSI) changes monthly (**a**) and different ovarian maturation stages (**b**) of blue tang fish (Box–Whisker diagram: +: Mean value; ●: Minimum or maximum value; *p* = 0.05).

The GSI varied by months during the year cycle, the rank was 0.08–0.96% and the average was 0.44 ± 0.22%. The GSI significantly increased from March to the period of April–July when the ovary of a fish was processed for maturation and ovulation and then decreased sharply after August to October (*p* < 0.05) (Figure 2). The GSI also significantly increased from maturity stages I to IV and decreased to stage V (*p* < 0.05).

### 3.2. Steroid Hormone Level at Different Ovarian Maturation Stages of Wild-Caught Blue Tang Fish

**Figure 3:**
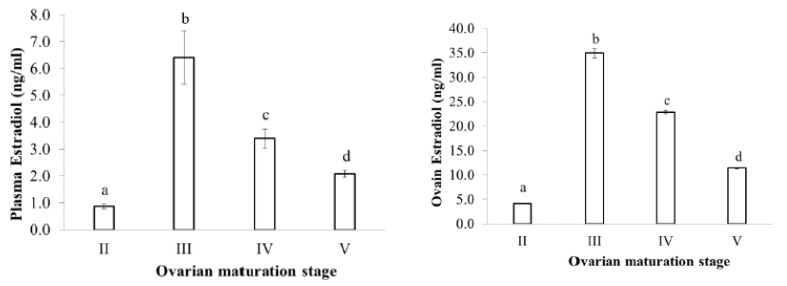
Estradiol level at different ovarian maturation stages of the blue tang fish (n the same as Figure 2b; data with a different subscript letter (a, b, etc.) are significantly different at *p* = 0.05).

**Figure 4:**
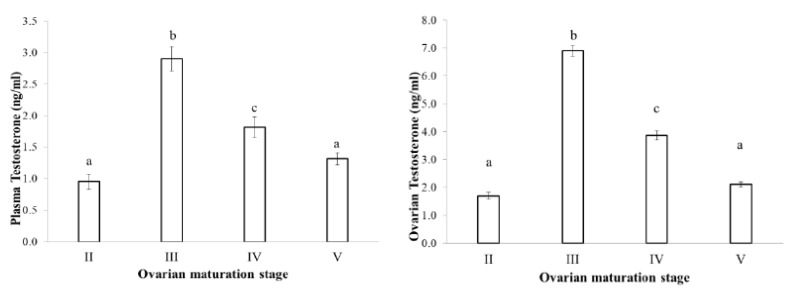
Testosterone level at different ovarian maturation stages of the blue tang fish (n the same as Figure 2b; data with a different subscript letter (a, b, etc.) are significantly different at *p* = 0.05).

**Figure 5:**
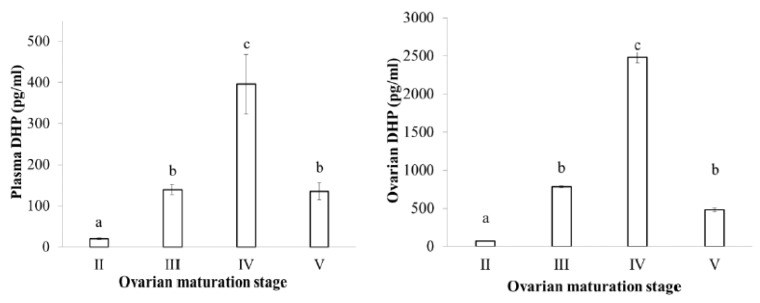
DHP level at different ovarian maturation stages of the blue tang fish (n the same as Figure 2b; data with a different subscript letter (a, b, etc.) are significantly different at *p* = 0.05).

Estradiol levels in plasma and ovaries have similar changes, low in the II stage, reaching the peak in the III stage and decreasing in the latter stages when fish undergo spawning (Figure 3). Testosterone levels in plasma and ovaries were also low in the II stage, high in the III stage and decreased in the latter stages IV and V (Figure 4). DHP levels in plasma and ovaries were remarkable in the III stage, especially increased in the IV stage, when fish were undergoing spawning (Figure 5).

### 3.3. Monthly Variation in Steroid Hormone Levels of Wild-Caught Blue Tang Fish

**Figure 6:**
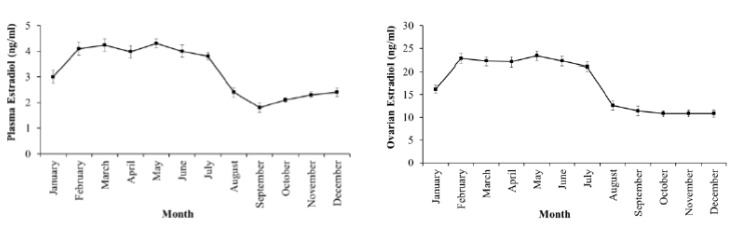
Monthly change in estradiol level in plasma (**left**) and ovarian (**right**) of the wild-caught blue tang fish (n the same as Figure 2a).

**Figure 7:**
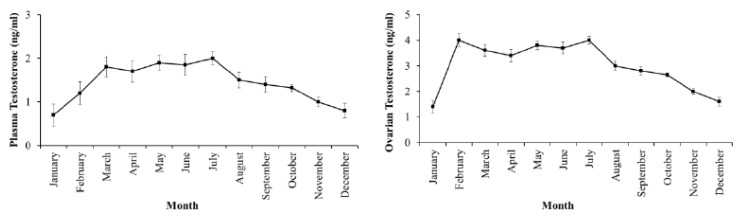
Monthly change in testosterone levels in plasma (**left**) and ovaries (**right**) of the wild-caught blue tang fish (n the same as Figure 2a).

**Figure 8:**
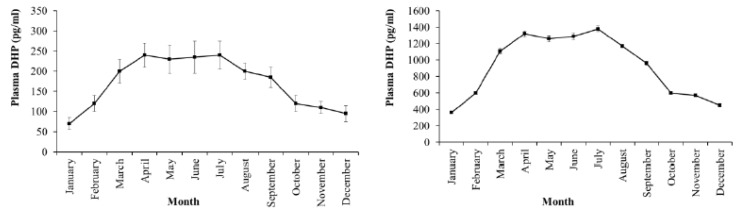
Monthly change in 17α, 20β-dihydroxy-4-pregnen-3-one (DHP) levels in plasma (**left**) and ovaries (**right**) of the wild-caught blue tang fish (n the same as Figure 2a).

Estradiol levels in plasma and ovaries varied by month during the year cycle. The period from February to July with the levels in plasma (average of 3.80–4.30 ng/mL) and ovaries (average of 22.20–23.40 ng/mL) significantly higher than the period from August to December (for plasma: Average of 1.80–2.40 ng/mL, and ovaries: Average of 10.80–12.60 ng/mL) of the next year (*p* = 0.05) when fish with ovaries in the III and IV stages were dominant from April to July (Figure 6). The level of testosterone in plasma and ovaries changed similarly to estradiol with months when higher values were found from March to July (*p* = 0.05) (Figure 7); in this period, the value of plasma averaged 1.7–2.0 ng/mL and of ovaries averaged 3.4–4.0 ng/mL. The higher level of DHP in plasma (average of 200–240 pg/mL) and ovaries (average of 1110–1380 pg/mL) was also observed to be considerable from March to August during the spawning period (*p* = 0.05) (Figure 8).

## 4. Discussion

Steroid hormones play a vital role in the regulation of oocyte growth, maturation and ovulation in female fish [1,14]. Some steroid hormones are involved in the sexual and spawning behavior of fish. Both E_2_ and T are produced in the ovaries of female teleosts. The ovarian two-cell model synthesizes E_2_ and T, where the theca cells synthesize T, which is consequently aromatized by cytochrome P450 aromatase (CYP19) to E_2_ by the granulosa cells [15]. E_2_ is responsible for vitellogenesis in female fish through the activation of vitellogenin (Vtg) and eggshell Zr-protein formation in liver. From the liver, Vtg is secreted into blood, transported to the ovary and absorbed into maturing oocytes. In addition to a precursor for E_2_ synthesis, T can enhance stimulatory effects of gonadotropins (GTHs) in vitro [16] and may also contribute to oocyte growth through the initiation of germinal vesicle break down (GVBD) during final oocyte maturation [17]. Similar to other teleosts, in blue tang, variation in the level of these hormones could be used as an indication of reproductive status in fish. Before the reproducing season, most oocytes in the chromatin nucleolus and perinucleolus stages existing in the ovary of blue tang with a low value of GSI implicated that the ovary of a fish was still inactive; during this period, the levels of T and E_2_ were low in both plasma and the ovary of the fish. In protandrous seabass, fish in the transitional stages showed low concentration for the steroids T, 11-KT, E_2_ and estrone in the plasma, whereas 11β-hydroxyandrostenedione was the most abundant androgen in testes and exhibited low in ovaries [18]. Regardless of the sex type, the T and 11-KT levels in gonads were not significantly different, whereas there were higher levels in vitellogenin and atretic ovaries. In the sex-changing fish, the androgens of T, 11-KT and rostenedione and 11β-hydroxyandrostenedione and the estrogen E_2_ were low/extreme low levels both in testes and ovaries, but the estrogen E_2_ was relatively upregulated in transitional gonads from the early stage of sex reversal [18]. In wild female Japanese flounder and barfin flounder-multiple spawners, plasma levels of T and E_2_ were observed at a very low value in the fish with oocytes in the chromatin nucleolus, perinucleolus and previtellogenic stages [19,20,21]. Similar to the above-mentioned cases, it is possible to assume that the endocrinology system in reproduction is inactive in the early stages of ovarian growth in blue tang.

The increase of the T and E_2_ levels in plasma and ovaries in the III stage with the increase in size of ovaries and GSI should be considered as an implementation of maturational initiation in the blue tang in early spawning season. The increase of these steroids could be associated with the increase in the water temperature and day length during this time. In wild Japanese flounder, the T and E_2_ levels in plasma were elevated in fish at the onset of the spawning season [19]. In female tilapia *Oreochromis mossambicus* [22], captive female barfin flounder [20] and chub mackerel [23], plasma levels of T and E_2_ increased in accordance with the increased GSI in the vitellogenic fish. While the role of T in female teleosts remains unclear, T may have vitellogenic action of its own at high concentrations [24] and may perform a role in maintaining oocytes once vitellogenesis is complete. T is the precursor of E_2_ which stimulates vitellogenin synthesis [25] due to conversion of T into E_2_ by the enzyme aromatase. E_2_ levels have a significant relation with GSI and oocyte size during the period of vitellogenesis. Change in levels of these steroids in blue tang is similar to that in reared female Asian seabass [18], wild female Japanese flounder [19] and barfin flouder [20] in which plasma concentrations of T and E_2_ peaked during vitellogenesis during the reproductive cycle in a year. In general, E_2_ is secreted by both the female gonads and inter-renal tissues in teleosts. E_2_ in blue tang is also buried by female gonads during the pre-spawning period and responsible for stimulating vitellogenesis.

In blue tang fish, the study found that T and E_2_ levels in plasma and ovary declined toward the late period of the spawning season when the fish experiences the regression phase, similar to the red seabream [26], turbot [27], barfin flounder [20] and Japanese flounder [19]. A decrease in steroids in these fish could reflect that activity of the hypothalamic–pituitary–gonadal axis was diminishing at the end of the spawning season.

DHP—a common maturation-inducing hormone (MIH) in most teleosts, which plays a critical role for GVBD and prepares the oocyte for successful fertilization—is induced by GTHs [22,28]. In blue tang, DHP levels could be detected in all stages of ovarian development, but a high level was found in early spawning fish. The increase of plasma DHP levels throughout oocyte maturation could support the role of DHP as the MIH in female blue tang. In addition, toward the end of the spawning season, although T and E_2_ levels were low, DHP levels were still considerable in most females. This result could reflect reproductive status during this period, when activity of oocyte recruitment was diminishing, while final oocyte maturation and spawning activity were still progressing. Consequently, a high DHP level can be necessary for final oocyte maturation and ovulation in female blue tang. In fish, some C-21 steroids, such as DHP and 17α, 20β, 21-trihydroxy-4-pregnen-3-one (20β-S), regulate the final maturation of the oocytes and ovulation or spermiation [1]. Among them, DHP is a common MIH in the majority of teleosts investigated [3,29,30]. However, whether DHP is the MIH in blue tang needs to be identified.

## 5. Conclusions

In summary, this study showed the fluctuation of the steroid hormone levels in plasma and ovaries in relation to ovarian maturation during a year in the wild female blue tang. These results also partly elucidate the roles of steroid hormones in the reproductive cycle of this species. Further study on the roles of GnRH-a in the gonadal maturation of blue tang fish in capture conditions should be conducted for the artificial breeding technique of this fish.

## Figures and Tables

**Table 1:** Maturity stages of female blue tang fish [9].

Maturity Stages	Particular of the Gonads
Stage I, Immature	Ovary was thin, short and glassy in appearance. The ovary and testes could not be distinguished by naked-eyes. The stage was observed in the fish size under the first maturation size.
Stage II, Maturing	Ovary developing; ovary and testes can be distinguished by with the naked-eye. Ovaries are opaque and creamy yellow.
Stage III, Mature	Ovaries increased in size compared to stage II. Ovaries are reddish yellow, extending about 2/3 body cavity length.
Stage IV, Ripe/Oozing	Ovaries are yellow to amber colored filling the entire body cavity, extending the entire body cavity length
Stage V, Spent	Ovaries are rather flaccid, reddish-yellow.
